# Effect of cognitive-behavioral therapy on fatigue in cancer patients: a systematic review and meta-analysis

**DOI:** 10.3389/fpsyg.2024.1435110

**Published:** 2025-01-10

**Authors:** Parisa Hosseini Koukamari, Mahmood Karimy, Mohtasham Ghaffari, Alireza Milajerdi

**Affiliations:** ^1^Social Determinants of Health Research Center, Saveh University of Medical Sciences, Saveh, Iran; ^2^School of Public Health and Safety, Shahid Beheshti University of Medical Sciences, Tehran, Iran; ^3^Research Center for Biochemistry and Nutrition in Metabolic Diseases, Institute for Basic Sciences, Kashan University of Medical Sciences, Kashan, Iran

**Keywords:** cancer, CBT, cancer-related fatigue, cognitive behavioral therapy, CRF, fatigue

## Abstract

**Background:**

Fatigue is a prevalent issue among cancer patients. Cognitive behavioral therapy (CBT) is an individualized intervention that empowers patients and caregivers to actively participate in the treatment process. A recent systematic review and meta-analysis examined the impact of CBT on fatigue in cancer patients.

**Methods:**

Articles published up to April 2023 were systematically searched in Scopus, PubMed, and the Web of Science using relevant keywords. All randomized clinical trials (RCTs) investigating the effects of CBT on fatigue in cancer patients were included. Statistical analyses were conducted using Stata version 14, with a significance level set at a *p*-value of <0.05.

**Results:**

The current systematic review and meta-analysis encompasses 10 RCTs. CBT demonstrated a significant reduction in fatigue scores among cancer patients [standardized mean difference (WMD): −2.50; 95%CI: −3.43, −1.56; I2 = 95.8%, *p* < 0.001]. This effect was consistent across all subgroup analyses.

**Conclusion:**

This study underscores the significant impact of CBT on fatigue in cancer patients. Further randomized clinical trials focusing on various cancer types are warranted to validate and build upon these findings.

## 1 Background

Cancer poses a significant public health challenge worldwide (Sun et al., 2023; Milajerdi et al., 2018). Global cancer statistics project that the number of cancer cases will soar to 27 million individuals by 2040 (Sung et al., 2021). The World Health Organization (WHO) reports that cancer contributes to the highest burden of diseases globally, accounting for a total of 244.6 million disability-adjusted life years (DALYs) worldwide (Mattiuzzi and Lippi, 2019). While advanced treatment modalities such as chemotherapy and radiotherapy have led to improved survival rates among cancer patients in recent years (Choi et al., 2022; Darooghegi Mofrad et al., 2020), the adverse effects of these treatments remain a major concern for public health systems (Sung et al., 2021). Cancer-related fatigue (CRF) is a prevalent issue among cancer patients, often persisting even after conventional cancer treatments (Liu et al., 2022). Patients endure a distressing and enduring sensation of physical, cognitive, and emotional fatigue linked to cancer or its treatment, disrupting their daily functioning (Kröz et al., 2023).

Given the increased longevity of cancer patients, enhancing their quality of life and survival outcomes while mitigating physical, psychological, and psychosocial challenges is paramount (Zhang et al., 2022). Psychosocial interventions such as cognitive behavioral therapy (CBT) have demonstrated effectiveness in alleviating various physical and mental complications associated with cancer (Moorey and Greer, 2011). CBT interventions encompass a range of psychotherapeutic techniques aimed at addressing maladaptive behaviors and psychological distress through cognitive and behavioral modifications (Kaplan et al., 1995). By imparting essential skills to modify dysfunctional thoughts and behaviors, CBT empowers patients to take control of their condition, thereby boosting their self-assurance in battling the disease (Grégoire et al., 2017; Dobson and Dozois, 2021). A systematic review and meta-analysis focusing on breast cancer patients revealed that CBT significantly enhanced the quality of life among this patient population (Getu et al., 2021; Yennurajalingam et al., 2023). Furthermore, a systematic review evaluating the efficacy of CBT in managing cancer-related fatigue (CRF) in advanced cancer patients demonstrated a significant reduction in CRF scores compared to standard care (Poort et al., 2020). Another meta-analysis involving patients undergoing or post-cancer treatment indicated that combined CBT effectively reduced CRF levels (Hilfiker et al., 2018). Consistent findings were also observed in a comprehensive analysis of 6 randomized controlled trials (RCTs) involving 472 breast cancer patients (Zhang et al., 2019).

Despite these insights, to the best of our knowledge, there exists no comprehensive systematic review and meta-analysis specifically investigating the effects of CBT as a standalone intervention on CRF in cancer patients. Therefore, our objective is to conduct a current systematic review and meta-analysis to explore the impact of CBT on fatigue in individuals with cancer.

## 2 Methods

This systematic review and meta-analysis was performed according to PRISMA guidelines (Page et al., 2021). Scopus, PubMed, and Web of Science were searched using MESH and non-MESH terms up to April 2023: ((“cognitive therapy”[tiab] OR “cognitive-behavioral therapy”[tiab] OR “Cognitive Behavioral Intervention”[tiab] OR “cognitive behavioral therapy”[tiab] OR CBT[tiab]) AND (“cancer”[tiab] OR “tumour”[tiab] OR “tumor” [tiab]) AND (trial[tiab])). No time or language restrictions were applied during the search.

### 2.1 Eligibility criteria

The following criteria were considered for including studies: (A) original randomized controlled clinical trials, (B) studies conducted among cancer patients, (C) studies investigating the effects of cognitive-behavioral therapy alone, and (D) reported changes in fatigue score or baseline and final scores of fatigue in the intervention and control groups.

The exclusion criteria were: (1) systematic reviews and meta-analyses, (2) studies involving healthy subjects or patients with other diseases than cancer, (3) studies without a control group, and (4) studies examining multiple interventions along with CBT. In addition, grey literature, including theses and conference abstracts, was not included.

### 2.2 Data extraction

The following data were collected from each study by two independent researchers: first author’s name, year of publication, study location, study design, mean age and standard deviation, gender distribution in the intervention and control groups, number of participants in each group, intervention and control groups, mean and standard deviation of fatigue score before and after the intervention in each group, and any adjustments made for confounding factors. To ensure the reliability and accuracy of the data, two researchers independently collected them. Any disagreements between the researchers were resolved by a third researcher (AM).

### 2.3 Statistical analysis

Mean differences and confidence intervals were calculated using mean and standard deviation (SD) changes in the fatigue score between the intervention and control groups. In cases where variation was reported in other variables than SD, we calculated SD using the appropriate formula. Between-study heterogeneity was assessed using the Cochrane Q test and I^2^ statistics, which quantify the degree of variability across studies included in the analysis. In cases of significant between-studies heterogeneity, random-effect analysis was used for the analysis. Subgroup analyses were performed using the fixed-effect model. Stata version 14 (Stata Corp., College Station, TX) was used in the current meta-analysis. A *p*-value of <0.05 was considered statistically significant.

## 3 Results

**Figure 1:**
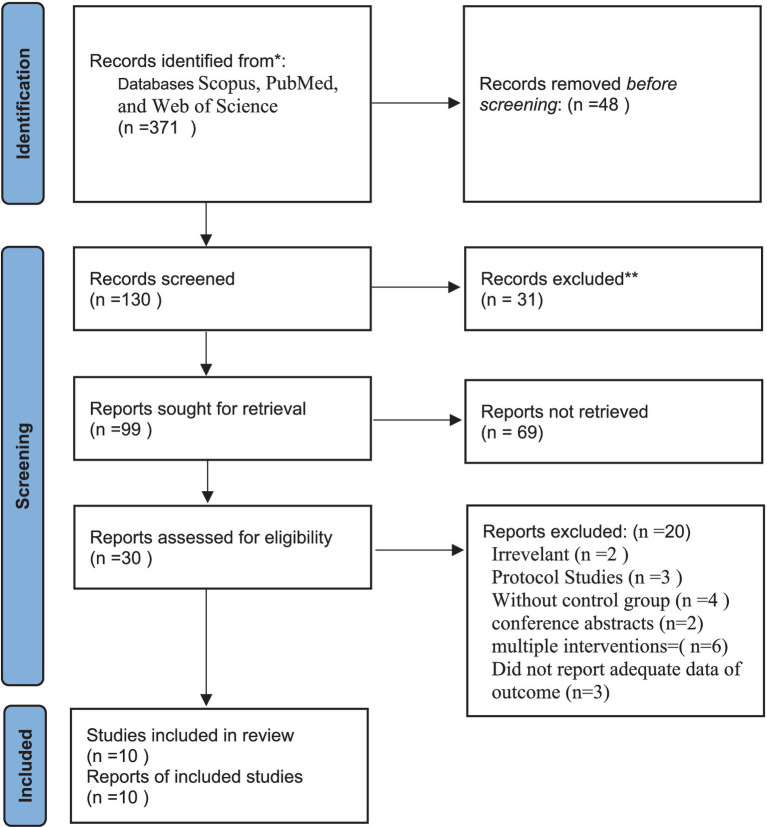
Flow diagram demonstrates the study selection process.

Overall, 371 papers were found in our initial search. After initial screening by title and abstract, 56 articles remained for the second stage of screening. Some of these articles were excluded after screening by the full text. In total, 10 RCTs were included in the current systematic review and meta-analysis (Grégoire et al., 2017; Abrahams et al., 2017; Bean et al., 2021; Goedendorp et al., 2014; Goedendorp et al., 2010; Hyland et al., 2022; Jim et al., 2020; Sheikhzadeh et al., 2021; van Weert et al., 2010; Wells-Di Gregorio et al., 2019). The flow diagram of study selection is shown in Figure 1.

### 3.1 Characteristics of included studies

The general characteristics of included studies are summarized in Table 1. The studies were published between 2010 and 2022. Overall, 754 cancer cases participated in those studies. Four studies were performed exclusively on women (Grégoire et al., 2017; Abrahams et al., 2017; Bean et al., 2021; Jim et al., 2020), while the others included both genders. Cognitive behavioral therapy (Grégoire et al., 2017; Abrahams et al., 2017; Bean et al., 2021; Goedendorp et al., 2014; Jim et al., 2020; Sheikhzadeh et al., 2021; Wells-Di Gregorio et al., 2019) and physical training combined with cognitive-behavioral therapy (Goedendorp et al., 2010; Hyland et al., 2022; van Weert et al., 2010) were their interventions. Usual care (Abrahams et al., 2017; Goedendorp et al., 2010), physical training alone (van Weert et al., 2010), and being put on the waiting list (Goedendorp et al., 2010; Hyland et al., 2022; Jim et al., 2020; Sheikhzadeh et al., 2021; Wells-Di Gregorio et al., 2019) were the control groups. The study duration ranged between 6 weeks and 6 months.

**Table 1:** General characteristics of included studies.

ID	Code/Author (year)	Subjects and gender	Age range (y) And mean	Design	Intervention type		Duration (wk/d)	Outcomes	Outcome assessment method	Outcome	
					Intervention (name and composition)	Control (name and composition)				Intervention mean ± SD and number	Control mean ± SD and number
1	Abrahams et al. (2017)	*F* = 132 M = 0 Both= 132 ICBT = 61 CAU = 64	ICBT= 52.5 (8.2) CAU= 50.5 (7.6)	Parallel	Internet-Based Cognitive Behavioral Therapy	Care as usual	6wk	Fatigue severity	Fatigue severity: CIS-Fatigue Severity [range 8–56]	CIS-Fatigue Severity Baseline (mean ± SD)= 45.2 (7.0) 6 mo = 27.7 (12.2)	CIS-Fatigue Severity Baseline= 44.9 (7.5) 6mo= 39.1 (11.3)
2	Bean et al. (2021)	CBT+ *F* = 32 TAU+= F = 23	CBT + =48.52 (9.51) TAU+ = 51.19 (12.02)	Parallel	CBT+ CBT+ comprised one 60-min face-to-face	Treatment as usual with relaxation audio (TAU+)	6wk	Fatigue	Patient-Reported Outcomes Measurement System (PROMIS) scales	Fatigue Change Baseline to Post = −4.67 (−6.98, −2.35)	Change Baseline to Post = −0.49 (−2.91, 1.93)
3	Goedendorp et al. (2014)	CBT (*n* = 50) M = 27 F = 23 waiting list (WL) control condition (*n* = 48) M = 23 F = 25	CBT = 44.6 (9.9) WL= 45.3(10.3)	Parallel	Cognitive Behavioral Therapy	Waiting List	6 month	Fatigue	Fatigue Severity subscale of CIS	CIS-Concentration CBT Baseline (mean ± SD) = 24.4 (7.8) T2: 17.6 (8.9)	CIS-Concentration wl Baseline = 22.9 (9.7) T2: 20.1 (8.5)
4	Goedendorp et al. (2010)	CBT *n* = 76 F = 48 M = 28 UC *n* = 72 M = 25 F = 47	CBT= 55.6 (11.3) UC= 57.3 (11.1)	Parallel	Participants in the CBT group received up to ten 1-h sessions during 6 months	Usual case	6 months	Fatigue severity	Fatigue subscale of the Checklist Individual Strength (CIS)	CIS-fat CBT (mean ± SD) = T1= 25.3 (14.0) T2= 21.0 (11.6)	CIS-fat UC= T1 = 23.4 (12.4) T2= 25.9 (13.5)
5	Grégoire et al. (2017)	CBT (N = 10) Control= (*N* = 24)	CBT= 53.2 (12.4) Control= 52.5 (6.8)	Parallel	Cognitive behavioral therapy intervention included 6 weekly 90 min sessions in groups of 3–8 participants led by CBT-trained psychologists with experience in psycho-oncology	The control group included patients who agreed not to attend any of the groups.	6 wk	Fatigue	European Organization for Research and Treatment of Cancer QoL Core Questionnaire-30	CBT T0 (mean ± SD) = 2.20 (0.65) T1 = 2.07 (0.49)	Control T0 = 2.56 (0.92) T1 = 2.36 (0.74)
6	Hyland et al. (2022)	CBT-TTF (*N* = 22) WLC (*N* = 14)	CBT-TTF 53.7 (11.0) WLC 59.6 (12.4)	–	Cognitive behavioral therapy for targeted therapy related fatigue	Waitlist control condition	18 wk	Fatigue catastrophizing	Fatigue Catastrophizing Scale (FCS)	Fatigue catastrophizing (FCS) Baseline = 13.7 (6.5) Follow-up = 4.9 (6.3)	Fatigue catastrophizing (FCS) Baseline = 8.6 (5.4) Follow-up = 7.1 (5.8)
7	Jim et al. (2020)	CBT-TTF (n = 29) WLC (*n* = 15)	CBT-TTF= 53 (13) WLC= 60 (12)	Parallel	Cognitive behavioral therapy for targeted therapy-related fatigue	Waitlist control	18 wk	Fatigue	Fatigue subscale of the Functional Assessment of Chronic Illness Therapy– Fatigue (FACIT-F)	Baseline = 28.03 (6.92) Follow-Up = 15.70 (8.69)	Baseline = 24.53 (7.05) Follow-Up = 24.71 (8.18)
8	Sheikhzadeh et al. (2021)	CBT= *F* = 18 M = 1 WLG *F* = 14 M = 6	CBT = 40/10 WLG= 37/45	Parallel	Cognitive behavioral therapy	Wait-list group	2 month	Fatigue	Cancer- Related Fatigue Scale (CFS)	Fatigue CBT Pre-test = 25.36(8.07) Post-test= 16.52(7.70)	Fatigue Pre-test = 22.80(8.77) Post-test = 21.60(8.10)
9	van Weert et al. (2010)	PT + CBT Group *N* = 76 PT Group *N* = 71	PT + CBT= 47.8 (10.5) PT Group = 49.9 (11.3)	Parallel	Physical training combined with cognitive-behavioral therapy	Physical training alone (PT group)	12 wk	Fatigue	Fatigue was measured with the MFI	General fatigue Preintervention= PT + CBT = 15.7 (3.5) Postintervention= 11.4 (3.3)	General fatigue PT Preintervention = 15.6 (3.3)b Postintervention = 11.6 (3.8)
10	Wells-Di Gregorio et al. (2019)	Intervention (*n* = 17) Waitlist Control (*n* = 11)	Intervention= 55.59 (7.25) Waitlist Control= 58.0 (9.35)		Three-session acceptance-based cognitive behavioral -acceptance and commitment therapy (CBT-ACT) intervention	Waitlist control	6 wk	Fatigue	Fatigue Symptom Inventory (FSI)	Baseline (mean ± SE) = 26.12 (3.21) Week 6 = 20.86 (3.11)	Baseline = 20.82 (3.99) Week 6 = 17.73 (3.63)

### 3.2 Findings from the meta-analysis

**Figure 2:**
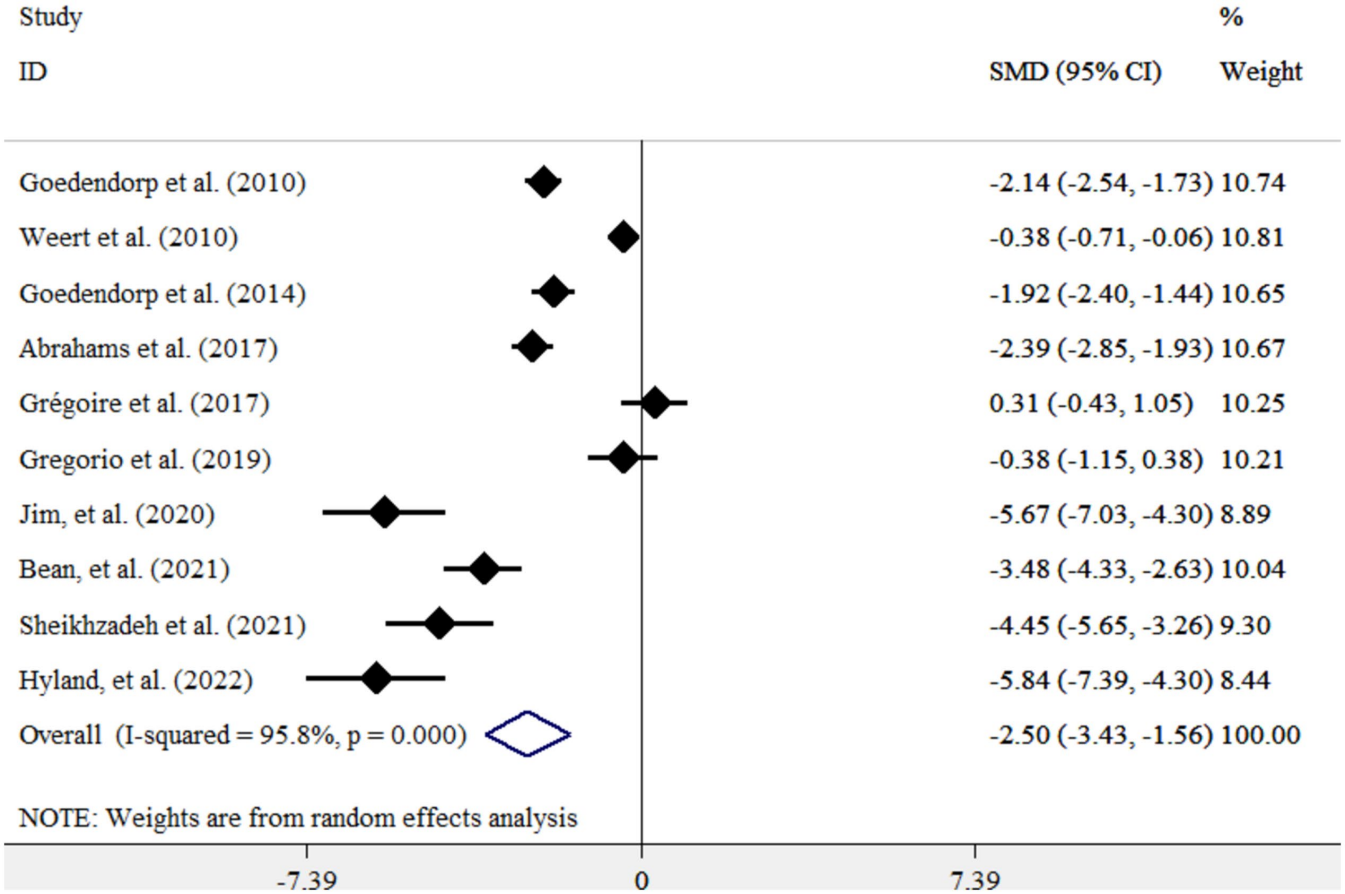
Forest plot for the effects of CBT on fatigue scores in cancer patients. Diamonds represent pooled estimates from random-effects analysis. Horizontal lines represent 95% CIs.

**Figure 3:**
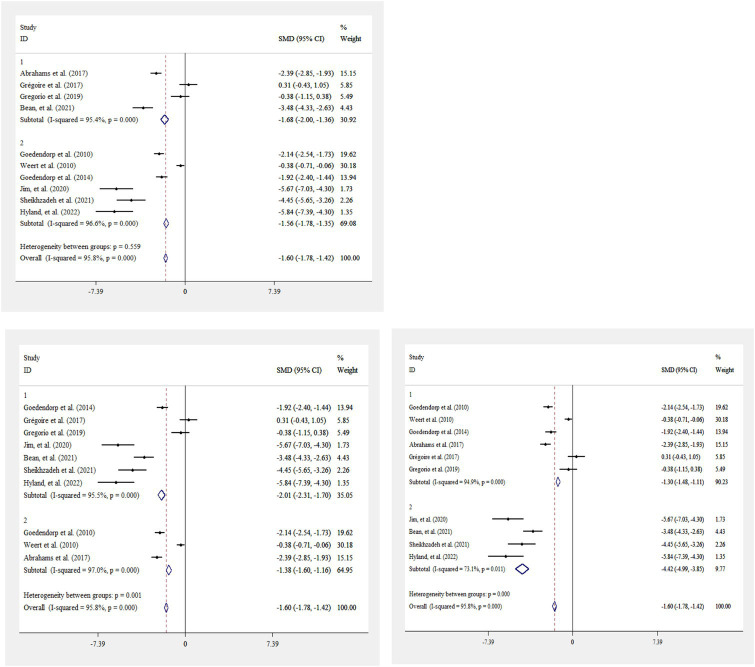
Forest plot for the effects of CBT on fatigue score in cancer patients, stratified by study duration (<1 month, ≥1 month) **(A)**, study sample size (<100, ≥100) **(B)**, and study publication year (<2020, ≥2020) **(C)**. Diamonds represent pooled estimates from random-effects analysis. Horizontal lines represent 95% CIs.

CBT resulted in a significant reduction in fatigue score in cancer patients [standardized mean difference (WMD): −2.50; 95%CI: −3.43, −1.56, I^2^ = 95.8%, *p* = <0.001] (Figure 2). This finding remained unchanged after subgroup analysis by study duration (<1 month, ≥1 month), study sample size (<100, ≥100), and the study publication year (<2020, ≥2020) (Figures 3A–C).

## 4 Discussion

The current study demonstrated a significant impact of cognitive behavioral therapy (CBT) on fatigue scores in cancer patients, even conducting a subgroup analysis. To the best of our knowledge, this represents the inaugural systematic review and meta-analysis delving into the effects of CBT as a standalone intervention on fatigue in individuals with cancer.

In alignment with our meta-analysis, a recent systematic review and meta-analysis also underscored the notable effect of CBT on fatigue (Xu et al., 2023). This study highlighted the efficacy of CBT-based interventions in reducing sick leave duration and facilitating employees’ return to work. Conversely, a separate meta-analysis focusing on patients with knee osteoarthritis failed to observe a significant enhancement in fatigue levels (Lin et al., 2023). While several systematic reviews have explored the impact of CBT in conjunction with other psychological interventions on fatigue and other mental or physical variables in cancer patients (Maunick et al., 2023; Belloni et al., 2022; Blumenstein et al., 2022), the heterogeneous nature of the included studies has precluded definitive conclusions regarding the effects of CBT on fatigue in this population.

CBT is a personalized intervention that empowers patients and caregivers to actively engage in the treatment process (Sensky et al., 2000). The low dropout rates associated with CBT underscore its acceptability among patients. This approach enables individuals to gain insights into their symptoms, develop more adaptive attributions, and cultivate coping strategies for various manifestations, such as auditory hallucinations and paranoid beliefs. Moreover, CBT has been shown to enhance attitudes toward treatment, promote medication adherence, reduce relapse rates, and lower re-hospitalization rates (Rathod et al., 2005; Gumley et al., 2003). Importantly, CBT is considered a safe therapeutic modality, with no documented elevations in suicidal ideation, agitation, or violent behaviors (Turkington et al., 2003).

This study marks the first meta-analysis specifically focusing on fatigue in cancer patients within the context of CBT as a standalone intervention. The analysis encompassed studies where CBT interventions were administered independently, and fatigue was assessed using validated methodologies. However, certain limitations should be acknowledged. The meta-analysis included diverse cancer types, suggesting the need for further randomized controlled trials tailored to each specific cancer type. Additionally, variations in the measurement of fatigue using different questionnaires, lack of adjustment for confounding variables in the majority of studies, and limited reporting on distinct types of fatigue hindered the ability to conduct subgroup analyses. Finally, discrepancies in the duration of interventions served as a potential source of bias in the current meta-analysis.

## 5 Conclusion

In conclusion, our systematic review and meta-analysis demonstrated a significant effect of CBT on fatigue in patients with cancer. Further randomized clinical trials focusing on various types of cancer are necessary to provide additional insights into this matter.

## Data Availability

The original contributions presented in the study are included in the article/supplementary material, further inquiries can be directed to the corresponding authors.
